# Role of Visceral Adipose Tissue in Predicting the Prognosis of Acute Pancreatitis: A Tertiary Level Hospital-Based Study From Chennai

**DOI:** 10.7759/cureus.101044

**Published:** 2026-01-07

**Authors:** Philson J Mukkada, Vinoth Thangam, Ganesh Gokul, Teenu Franklin

**Affiliations:** 1 Radiodiagnosis, Sri Venkateshwara Medical College and Hospital, Chennai, IND; 2 Radiodiagnosis, ACS Medical College and Hospital, Chennai, IND; 3 Radiology, ACS Medical College and Hospital, Chennai, IND; 4 Radiology, Scans World, Chennai, IND

**Keywords:** acute pancreatitis, apache ii, ct imaging, mctsi, vat/smt ratio, visceral adipose tissue

## Abstract

Background: Acute pancreatitis (AP) ranges clinically from mild, self-limiting inflammation to severe disease with multi-organ failure. Visceral adipose tissue (VAT), a key contributor to systemic inflammation and metabolic dysfunction, may therefore influence AP severity, yet its prognostic value in the Indian population remains limited in the literature.

Aim: The aim of this study is to assess whether VAT and the VAT/SMT (skeletal muscle tissue) ratio can predict clinical severity and short-term prognosis of AP in a tertiary care hospital in Chennai.

Materials and methods: This cross-sectional study included 62 patients aged 18-60 years diagnosed with AP at ACS Medical College and Hospital from December 2023 to February 2025. Contrast-enhanced CT scans obtained within 24 hours were used to quantify VAT, subcutaneous adipose tissue, SMT, and VAT/SMT ratios at the L3-L4 vertebral level. Severity grading was performed using APACHE II (Acute Physiology and Chronic Health Evaluation), modified computed tomography severity index (MCTSI), and the Revised Atlanta Classification. Statistical analysis was performed using IBM SPSS Statistics for Windows, Version 26 (Released 2018; IBM Corp., Armonk, New York, United States).

Results: Higher VAT values and elevated VAT/SMT ratios showed significant associations with increased APACHE II and MCTSI scores (p < 0.001). Patients with VAT >180 cm² and VAT/SMT >1.5 exhibited more severe disease patterns. The MCTSI demonstrated strong concordance with the Revised Atlanta Classification. Male sex with alcohol consumption was significantly associated with higher severity, while age showed no significant impact.

Conclusion: CT-derived VAT and VAT/SMT ratios are useful early markers associated with increased severity. Integrating CT-based VAT assessment with the MCTSI may enhance early risk stratification and guide timely clinical management.

## Introduction

Acute pancreatitis (AP) is an inflammatory condition of the pancreas with a clinical spectrum ranging from mild, self-limiting disease to severe forms associated with organ failure and significant mortality. A recent population-level analysis using data from 1990 to 2021 (via Global Burden of Disease Study, GBD) estimates that the age-standardized incidence rate of pancreatitis in India increased from ≈ 32.78 per 100,000 (1990) to ≈ 36.76 per 100,000 (2021) [1,2]. Although the diagnosis of AP is usually straightforward, predicting disease severity early remains challenging and is essential for guiding timely management.

Current classification systems, including the Revised Atlanta Criteria, categorize AP based on local complications and organ dysfunction, but a rapid, universally accepted, and cost-effective early prognostic marker is still lacking. Early identification of patients at risk of severe disease is crucial, as timely intervention during the initial phase can significantly reduce morbidity and mortality [3].

Obesity is an important modifiable factor known to worsen the course of AP. Visceral adipose tissue (VAT), in particular, contributes to a pro-inflammatory state, impaired microcirculation, and metabolic dysfunction, all of which may intensify pancreatic injury [4,5]. VAT has been implicated in the severity of several metabolic and cardiovascular diseases, yet its specific role in influencing AP outcomes remains insufficiently explored [6-8].

While CT is a reliable tool for quantifying VAT, the potential predictive value of VAT in determining AP prognosis has not been well established, especially within the Indian population, where variations in body fat distribution may influence disease behavior [9,10]. This study aims to evaluate the relationship between VAT and the clinical prognosis of AP in a tertiary care hospital in Chennai.

## Materials and methods

Study design and duration

This cross-sectional study was conducted at ACS Medical College and Hospital, Chennai from December 2023 to February 2025.

Study population and sample size

The study included 62 patients diagnosed with AP based on acute abdominal pain, elevated serum amylase or lipase, and radiological features consistent with AP. The sample size was determined using the standard formula for estimating proportions: sample size = (Z² × p × q) / d² derived from prevalence data by Ahmed et al. [1]. All additional eligible patients during the study period were also included.

Where Z is the standard normal deviate corresponding to a 95% confidence level (1.96), p is the expected incidence (0.36), q is 1 − p (0.64), and d is the allowable absolute error, taken as 13% (0.13).

A margin of error of 13% was selected due to the exploratory nature of the study, reliance on prior regional prevalence data, and feasibility constraints of early CT-based body composition analysis, which requires imaging within 24 hours, manual ROI segmentation, and dual-reader measurements; similar radiology-based studies in tertiary care settings have accepted margins of error up to 10-15%.

Thus, the minimum required sample size was 52 patients. To account for possible exclusions and incomplete data, and to improve the robustness of the analysis, all eligible patients during the study period were included, resulting in a final sample size of 62 patients.

Inclusion criteria

Patients aged 18-60 years presenting with a first episode of AP and referred for CT evaluation were included.

Exclusion criteria

Patients with a prior CT-confirmed history of pancreatitis or chronic pancreatic disease were excluded from the study.

Ethical committee approval

The ethical committee approval was obtained with ethical approval number (No.862/2023/IEC/ACSMCH) from the institutional Human Ethics Committee of ACS Medical College and Hospital, Chennai.

Data collection

Demographics

After obtaining institutional ethics approval and informed consent, patient demographics, BMI, etiology of pancreatitis, and clinical outcomes such as ICU admission, complications, and mortality were recorded.

Laboratory Testing

Laboratory parameters, including serum amylase, lipase, and creatinine, measured within 72 hours of admission were noted, and severity assessment was performed using APACHE II and the Revised Atlanta Classification.

Imaging

All patients underwent non-contrast-enhanced computed tomography (CT) imaging using a Siemens Somatom 32-slice scanner (M/ S Siemens Shenghai Medical Equipment Limited, Shenghai, China). Scans were acquired using standardized acquisition parameters within 24 hours of admission as it provides a baseline assessment of body composition before significant disease-related changes occur like acute inflammatory edema, fluid shifts, bowel ileus, and evolving peripancreatic collections which can alter fat and muscle attenuation and compromise accurate body composition measurements.

Axial images at the L3-L4 vertebral level were selected for body composition analysis.

**Figure 1:**
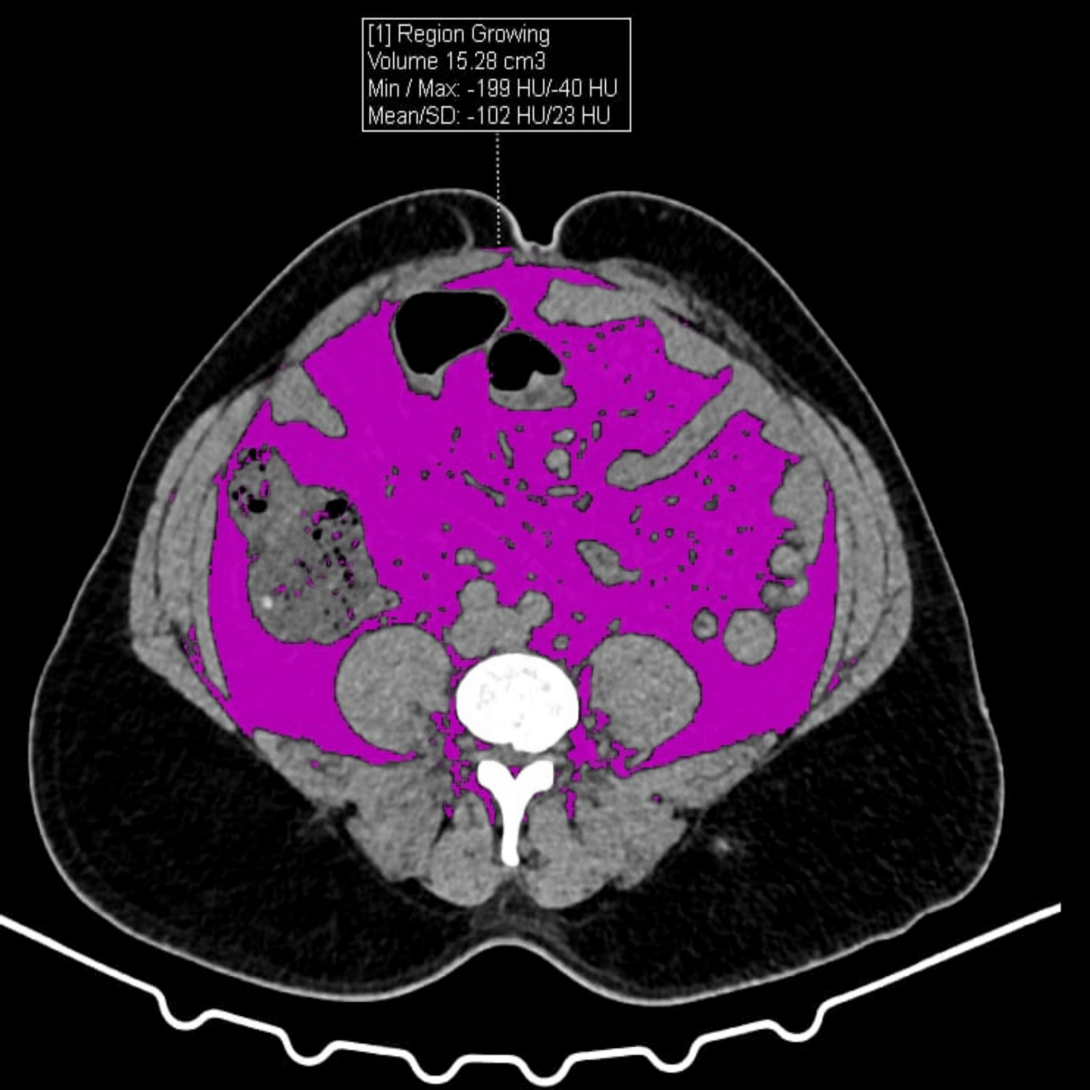
VAT calculations by CT within the ROI (region of interest) VAT: Visceral Adipose tissue

**Figure 2:**
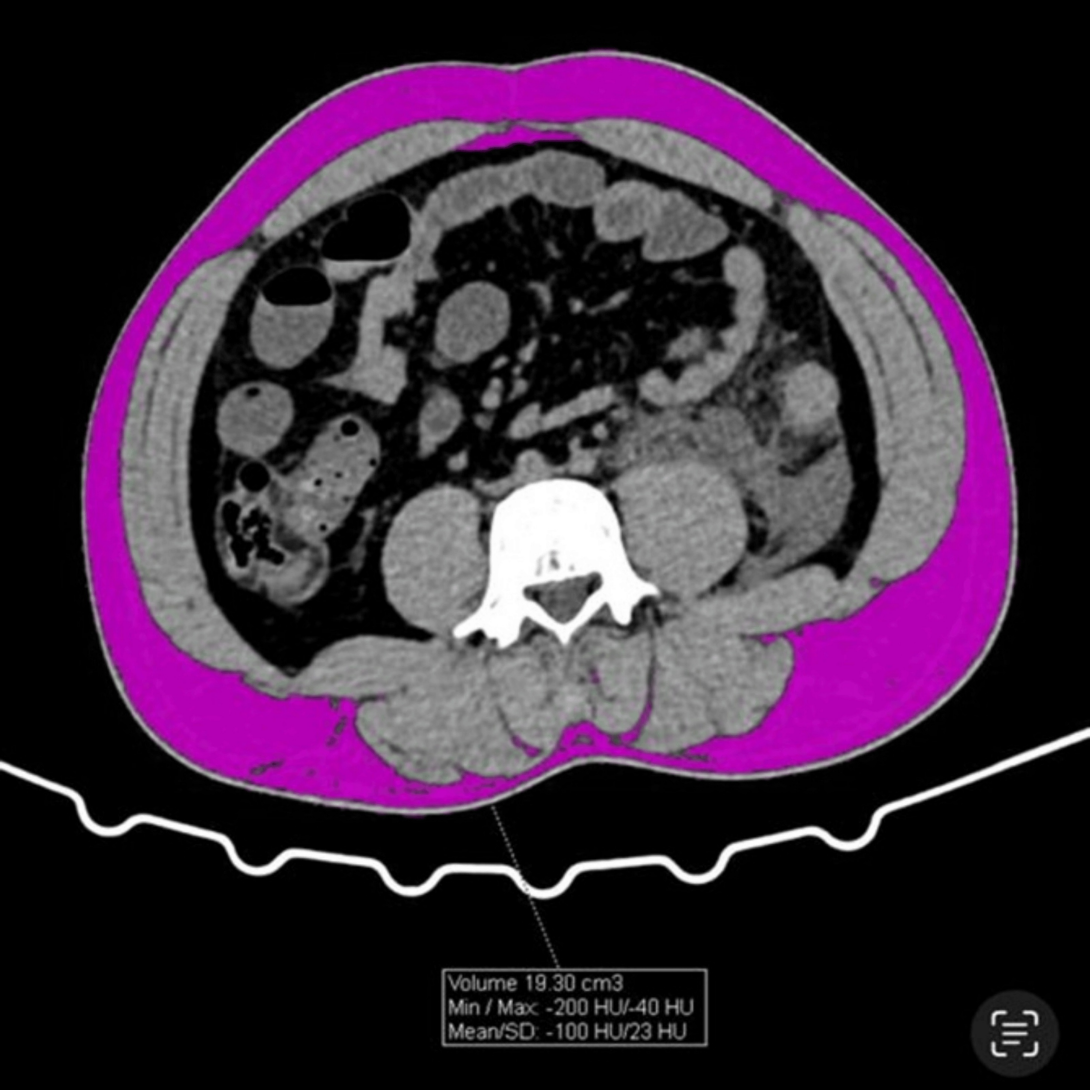
SAT calculations by CT within the ROI (region of interest) SAT: Subcutaneous Adipose tissue

**Figure 3:**
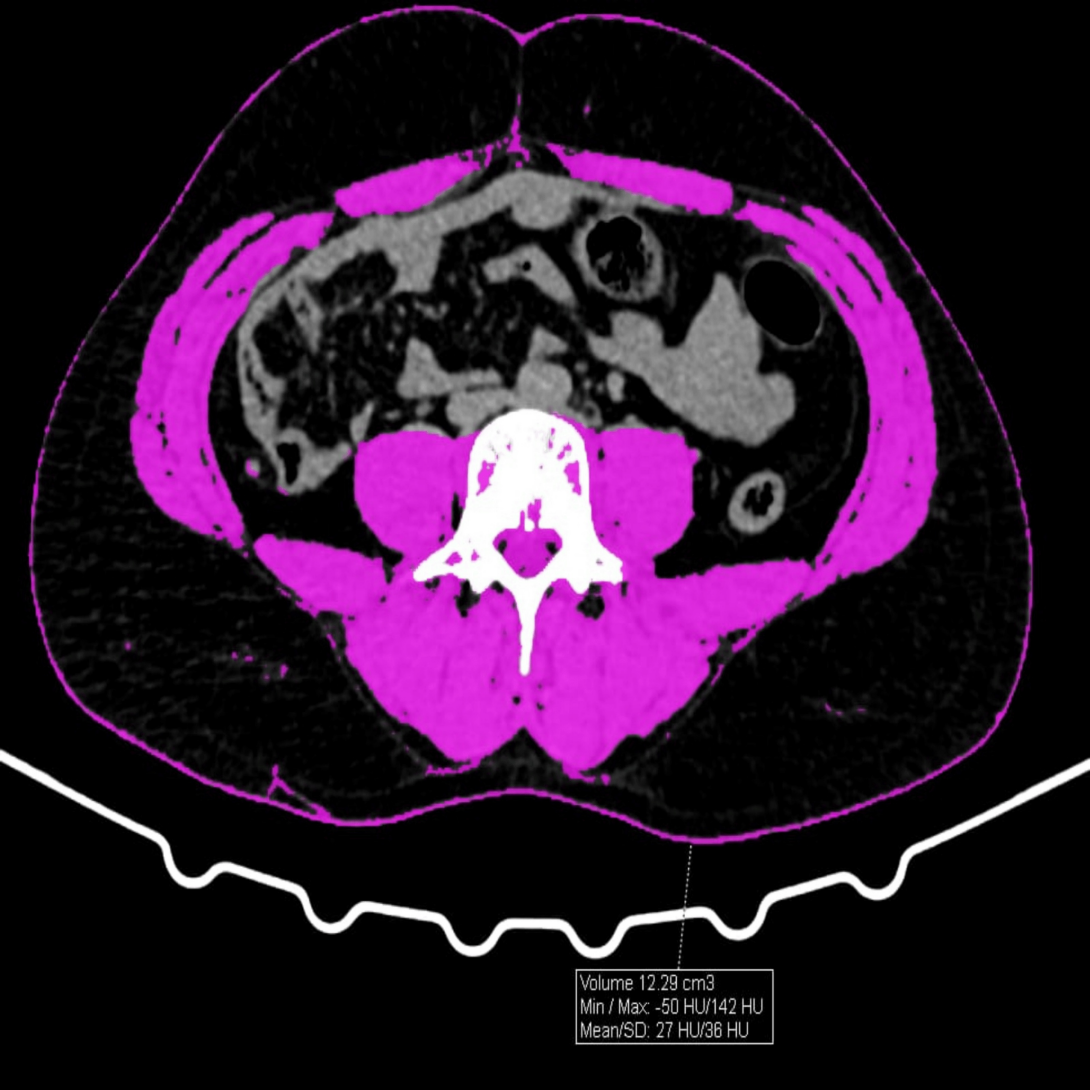
SMT calculations by CT within the ROI (region of interest) SMT: Skeletal Muscle Tissue

VAT, subcutaneous adipose tissue (SAT), and skeletal muscle tissue (SMT) areas were quantified by manual region-of-interest (ROI) segmentation on axial images using Adobe Photoshop CS6 following predefined attenuation and anatomical boundaries (Figures 1-3), as this approach demonstrated excellent inter-observer reliability (ICC > 0.85) and has been validated as comparable to dedicated software. The VAT/SMT ratio was subsequently calculated.

All measurements were independently performed by two blinded radiologists, and the mean of the two readings was used for final analysis. Inter-observer agreement was assessed using the intraclass correlation coefficient (ICC) to ensure measurement reproducibility.

The Modified CT Severity Index (MCTSI) was calculated to assess pancreatic inflammation, necrosis, and extra-pancreatic complications.

Patient confidentiality and privacy were maintained throughout the research process.

Statistical analysis

Data were extracted from hospital records, coded, and entered into Microsoft Excel, followed by statistical analysis using IBM SPSS Statistics for Windows, Version 26 (Released 2018; IBM Corp., Armonk, New York, United States). Welch’s t-test and Fisher's exact test were used. p<0.05 is considered statistically significant, and the findings were presented through tables and figures.

## Results

A total of 62 patients with AP were included in the study. The cohort was predominantly male and middle-aged, with a substantial proportion being overweight or obese (Table 1). Most patients presented with AP, and alcohol consumption was a common etiological factor. The majority did not require ICU care, and mild disease predominated based on both radiological (MCTSI) and clinical (Revised Atlanta Classification) severity grading (Table 2).

**Table 1:** Distribution of the participants according to their sociodemographic characteristics (n=62) BMI: Body mass index

Groups	Frequency (n)	Percent (%)
Age		
Below 30	13	21.0
31 - 39	29	46.7
40 - 49	15	24.2
50 and above	5	8.1
Total	62	100.0
Gender		
Male	47	75.8
Female	15	24.2
Total	62	100.0
	BMI (kg/m^2^)	
Normal (18.5 – 22.9)	27	43.5
Overweight (23 – 24.9)	15	24.2
Obese (>25)	20	32.3
Total	62	100.0

**Table 2:** Distribution of the participants according to their clinical history and clinical scores (n=62) MCTSI: Modified computed tomography severity index; ICU: intensive care unit

Groups	Frequency (n)		Percent (%)	
Diagnosis				
Acute Pancreatitis	52		83.9	
Acute on Chronic Pancreatitis	10		16.1	
Total	62		100.0	
Consumption of Alcohol History				
No	36		58.1	
Yes	26		41.9	
Total	62		100.0	
ICU Care				
No	56		90.3	
Yes	6		9.7	
Total	62		100.0	
MCTSI Score				
0		9		14.5
2		33		53.2
4		5		8.1
6		8		12.9
8		5		8.1
10		2		3.2
Total		62		100.0
MCTSI Score Interpretation				
Mild		42		67.7
Moderate		13		21.0
Severe		7		11.3
Total		62		100.0
Revised Atlanta Classification of AP (Clinically Used)				
Mild (MAP)		39		62.9
Moderately Severe (MSAP)		16		25.8
Severe (SAP)		7		11.3
Total		62		100.0

The mean age was 37.02 ± 8.15 years and mean BMI was 23.99 ± 2.96 kg/m². Mean MCTSI and APACHE II scores were 3.13 ± 2.60 and 5.65 ± 4.65, respectively. Mean SAT, VAT, SMT, and the VAT/SMT ratio were 129.57 ± 19.09 cm², 157.43 ± 40.16 cm², 141.73 ± 25.02 cm², and 1.19 ± 0.53, respectively, with mean serum amylase and lipase levels of 1213.69 ± 718.54 U/L and 5202.44 ± 4435.76 U/L (Table 3).

**Table 3:** Mean values of the variables of the study participants (n=62) Kg: Kilogram; m: meter; cm: centimeter; U/L: units per liter; BMI: body mass index; MCTSI: modified computed tomography severity index; APACHE: acute physiology and chronic health evaluation; VAT: visceral adipose tissue; SAT: subcutaneous adipose tissue; SMT: skeletal muscle tissue

Groups	Mean	SD
Age (years)	37.02	± 8.15
BMI (kg/m^2^)	23.99	± 2.96
MCTSI Score	3.13	± 2.60
APACHE II Score	5.65	± 4.65
SAT area(cm^2^)	129.57	± 19.09
VAT area (cm^2^)	157.43	± 40.16
SMT area (cm^2^)	141.73	± 25.02
VAT/ SMT	1.19	± 0.53
Amylase (U/L)	1213.69	± 718.54
Lipase (U/L)	5202.44	± 4435.76
Lipase/ Amylase Ratio	4.98	± 3.95

Body composition analysis demonstrated that increasing disease severity was associated with higher BMI, greater visceral and subcutaneous adipose tissue, and a progressively elevated VAT/SMT ratio, while SMT decreased significantly with severity (Table 4). These trends were consistent across both the Revised Atlanta Classification and MCTSI-based stratification. Physiological severity, as assessed by APACHE II scores, increased in parallel with rising adiposity and higher VAT/SMT ratios (Tables 4-6).

**Table 4:** Comparison of mean scores of variables under the subgroup of severity of AP based on the Revised Atlanta Classification Kg: Kilogram; m: meter; cm: centimeter; U/L: units per liter, BMI: body mass index; APACHE: acute physiology and chronic health evaluation; VAT: visceral adipose tissue; SAT: subcutaneous adipose tissue; SMT: skeletal muscle tissue

Group	Revised Atlanta Classification (Severity of AP)	Mean	SD	p-value
BMI (kg/m^2^)	Mild	22.43	1.89	< .001
	Moderately severe	25.54	2.25	
	Severe	29.16	0.33	
APACHE II Score	Mild	2.95	2.05	< .001
	Moderately severe	8.00	1.21	
	Severe	15.29	4.38	
SAT (cm^2^)	Mild	120.49	15.31	< .001
	Moderately severe	140.03	14.91	
	Severe	156.22	3.06	
VAT (cm^2^)	Mild	137.23	23.49	< .001
	Moderately severe	176.08	29.84	
	Severe	227.34	37.41	
SMT (cm^2^)	Mild	155.12	16.70	< .001
	Moderately severe	125.30	21.07	
	Severe	104.70	2.38	
VAT/ SMT	Mild	0.91	0.26	< .001
	Moderately severe	1.46	0.41	
	Severe	2.17	0.34	
Amylase (U/L)	Mild	1276.2	751.2	.089
	Moderately severe	1244.5	702.9	
	Severe	794.5	453.8	
Lipase (U/L)	Mild	3984.7	3260.0	.037
	Moderately severe	7865.3	6305.9	
	Severe	5900.2	2050.4	
Robust Tests of Equality of Means (Welch’s t-test); Significant p-value < 0.05				

**Table 5:** Comparison of mean scores of variables based on the VAT area Kg: Kilogram; m: meter; cm: centimeter; U/L: units per liter, BMI: body mass index; APACHE: acute physiology and chronic health evaluation; VAT: visceral adipose tissue; SAT: subcutaneous adipose tissue; SMT: skeletal muscle tissue

Group	VAT (cm^2^)	Mean	SD	p-value
BMI (kg/m^2^)	< 120	19.93	0.59	< .001
	121 - 150	22.24	0.78	
	151 - 180	24.71	0.58	
	> 180	28.00	1.20	
APACHE II Score	< 120	3.00	1.94	< .001
	121 - 150	2.90	2.07	
	151 - 180	5.00	3.05	
	> 180	11.38	4.55	
SAT (cm^2^)	< 120	99.26	3.90	< .001
	121 - 150	119.80	7.86	
	151 - 180	138.20	3.70	
	> 180	152.10	4.54	
SMT (cm^2^)	< 120	173.78	3.37	< .001
	121 - 150	159.93	5.00	
	151 - 180	132.38	8.71	
	> 180	108.30	4.51	
VAT/ SMT Ratio	< 120	0.60	0.04	< .001
	121 - 150	0.84	0.07	
	151 - 180	1.26	0.14	
	> 180	1.94	0.32	
Amylase (U/L)	< 120	1031.4	528.7	.490
	121 - 150	1419.4	869.2	
	151 - 180	1199.3	575.5	
	> 180	1084.8	739.6	
Lipase (U/L)	< 120	5654.3	5492.9	.107
	121 - 150	3650.5	2039.0	
	151 - 180	4613.5	3504.9	
	> 180	7448.8	5938.1	
Robust Tests of Equality of Means (Welch’s t-test); p-value significant if < 0.05				

**Table 6:** Comparison of variable mean scores using the VAT/SMT ratio Kg: Kilogram; m: meter; cm: centimeter; U/L: units per liter, BMI: body mass index; APACHE: acute physiology and chronic health evaluation; VAT: visceral adipose tissue; SAT: subcutaneous adipose tissue; SMT: skeletal muscle tissue

Group	VAT/ SMT Range	Mean	SD	p-value
BMI (kg/m^2^)	0.5 - 0.9	21.47	1.31	< .001
	1.0 - 1.5	24.63	0.49	
	> 1.5	27.88	1.25	
APACHE II Score	0.5 - 0.9	2.93	1.99	< .001
	1.0 - 1.5	4.67	2.84	
	> 1.5	11.29	4.42	
SAT (cm^2^)	0.5 - 0.9	112.95	11.92	< .001
	1.0 - 1.5	137.83	3.51	
	> 1.5	151.61	4.85	
VAT (cm^2^)	0.5 - 0.9	124.77	15.23	< .001
	1.0 - 1.5	165.26	8.52	
	> 1.5	208.17	29.56	
SMT (cm^2^)	0.5 - 0.9	164.55	7.99	< .001
	1.0 - 1.5	133.30	8.18	
	> 1.5	108.90	5.03	
Amylase (U/L)	0.5 - 0.9	1290.0	785.11	.609
	1.0 - 1.5	1230.2	581.82	
	> 1.5	1064.2	721.15	
Lipase (U/L)	0.5 - 0.9	4318.4	3607.0	.204
	1.0 - 1.5	4701.1	3609.8	
	> 1.5	7204.7	5836.9	
Robust Tests of Equality of Means (Welch’s t-test); p-value significant if < 0.05				

Visceral adiposity showed a strong, graded association with disease severity. Patients with higher VAT areas and VAT/SMT ratios had significantly higher APACHE II and MCTSI scores, indicating greater systemic involvement (Tables 5, 6). Skeletal muscle mass demonstrated an inverse relationship with both VAT and disease severity, suggesting a sarcopenic pattern in more severe cases. Serum lipase levels showed a significant association with severity, whereas amylase levels did not demonstrate consistent variation (Tables 4-6).

MCTSI scores were significantly higher in men, individuals with a history of alcohol consumption, and patients who developed local or systemic complications (Table 7). Increasing VAT and VAT/SMT ratios were strongly associated with higher MCTSI scores, further reinforcing the link between visceral adiposity and radiological severity. Age did not significantly influence disease severity (Table 7).

**Table 7:** Comparison of mean scores of MCTSI within different variables cm: centimeter; VAT: visceral adipose tissue; SMT: skeletal muscle tissue; MCTSI: modified computed tomography severity index

Category	Subcategory	N	MCTSI Score		p-value
			Mean	SD	
Age	Below 30	13	3.85	2.764	.778
	31 - 39	29	2.90	2.596	
	40 - 49	15	2.93	2.604	
	50 and above	5	3.20	2.683	
Gender	Male	47	3.49	2.71	.022
	Female	15	2.00	1.85	
H/O Alcohol Consumption	No	36	2.06	1.39	< .001
	Yes	26	4.62	3.13	
Local Complications	Nil	-	-	-	.010
	APFC	56	2.96	1.94	
	ANC	6	7.67	2.94	
Systemic Complications	No	43	1.63	0.90	< .001
	Yes	19	6.53	1.87	
Outcome	Death/ AMA	4	9.00	1.41	.070
	Treated	58	2.72	2.14	
VAT (cm^2^)	< 120	10	1.00	1.054	< .001
	121 - 150	20	1.80	.616	
	151 - 180	16	2.50	1.713	
	> 180	16	6.75	1.915	
VAT/ SMT Ratio	0.5 - 0.9	30	1.53	0.86	< .001
	1.0 - 1.5	15	2.27	1.48	
	> 1.5	17	4.67	1.86	
Robust Tests of Equality of Means (Welch’s t-test); p-value significant if < 0.05					

A strong concordance was observed between MCTSI and the Revised Atlanta Classification, particularly in mild and severe categories, with minimal discrepancy in moderate cases (p < 0.001), supporting the reliability of MCTSI as an imaging-based severity assessment tool that closely reflects clinical disease classification (Table 8). 

**Table 8:** Comparison of the MCTSI score and Atlanta Classification for grading the acute pancreatitis severity MCTSI: Modified computed tomography severity score

	Revised Atlanta Classification of Acute Pancreatitis				
MCTSI Score Interpretation		Mild	Moderately severe	Severe	Total
	Mild	38	4	0	42
	Moderate	1	12	0	13
	Severe	0	0	7	7
	Total	39	16	7	62
Fisher’s Exact Test; p-value < 0.001					

## Discussion

This study provides a comprehensive evaluation of AP severity in relation to sociodemographic characteristics, clinical history, body composition parameters, and biochemical markers, with particular emphasis on visceral adiposity and its association with disease outcomes. The analysis encompassed 62 participants from a South Indian population, and the findings offer important insights into the interplay between patient characteristics, imaging-based severity indices, and metabolic factors.

Sociodemographic profile and body mass index

The study population demonstrated a male predominance (75.8%) with most patients clustered in the 31-39-year age group (46.7%). This distribution aligns with prior reports indicating higher AP incidence among younger adult men, likely related to lifestyle factors such as alcohol consumption and dietary habits. The BMI distribution indicates that while 43.5% of participants were within the normal range, a substantial proportion were overweight (24.2%) or obese (32.3%), reflecting the growing prevalence of obesity in South Indian populations. Obesity, particularly central adiposity, has been consistently linked with increased risk of severe AP and poorer outcomes, likely due to pro-inflammatory adipokine activity and the metabolic impact of visceral fat.

Gender- and age-related body composition changes

Expected age and gender related variations in body composition were observed. Male patients exhibited higher BMI, SAT, and VAT values, consistent with their greater tendency for central fat accumulation. Subcutaneous fat increased and skeletal muscle mass declined with advancing age, reflecting physiological body composition changes. However, age and gender did not independently influence APACHE II scores or pancreatic enzyme levels. Previous studies have demonstrated gender and age-related differences in fat distribution, with men exhibiting greater central (abdominal) adiposity and women storing fat in the hips and thighs. Subcutaneous fat increases and skeletal muscle mass declines with age. In line with prior research, male patients in this study accounted for 75% of cases and had higher BMI, SAT, and VAT than female patients [11-15]. The study conducted by Lankisch et al. has similar findings, reporting that the male gender was affected more than the female population [16].

Clinical profile and etiology of acute pancreatitis

The majority of participants (83.9%) were diagnosed with AP, with 41.9% reporting alcohol use, highlighting its role as a key etiologic factor. Only 9.7% required ICU care, reflecting predominantly mild disease based on MCTSI (67.7%) and Revised Atlanta Classification (62.9%). Male predominance may be related to alcohol-related AP, while obesity further contributes to disease severity, consistent with previous studies [16,17].

Body composition parameters and biochemical markers

The mean age was 37 years, and the mean BMI was 23.99 kg/m², indicating moderate metabolic risk. Body composition analysis showed mean SAT 129.57 cm², VAT 157.43 cm², and VAT/SMT ratio 1.19, reflecting central adiposity. VAT promotes inflammation and metabolic dysregulation, potentially worsening pancreatic injury. Amylase (mean 1213.69 U/L) and lipase (mean 5202.44 U/L) were elevated but did not consistently correlate with disease severity.

Association of body composition with disease severity

Severe AP was associated with higher BMI, APACHE II scores, SAT, VAT, and VAT/SMT ratios, and lower SMT (p < 0.001), highlighting the impact of visceral adiposity and sarcopenic obesity on disease severity. Amylase levels showed no significant differences, while lipase was modestly elevated in moderately severe cases.

Impact of visceral adiposity on severity indicators

Increasing VAT was associated with higher BMI, APACHE II scores, SAT, and VAT/SMT ratios, while SMT declined (p < 0.001), indicating greater clinical severity with visceral adiposity. Patients with VAT >180 cm² showed the highest severity markers, suggesting a threshold effect. Amylase and lipase did not vary significantly. These findings align with prior studies confirming VAT and VAT/SMT as predictors of severe AP [10,18-22].

VAT/SMT ratio as a composite severity marker

Higher VAT/SMT ratios were strongly associated with increased BMI, APACHE II scores, SAT, and VAT, and decreased SMT (p < 0.001). Ratios >1.5 identified patients with worse clinical and metabolic profiles, highlighting VAT/SMT as a superior marker of central obesity and AP severity.

Role of MCTSI in severity assessment and prognostication

Higher MCTSI scores are significantly associated with an increased risk of infection and more severe local and systemic complications. Previous studies have shown that MCTSI is useful for identifying infectious complications; however, Liao et al. and Sahu et al. reported that it is less effective than APACHE II in predicting persistent organ failure [23,24]. Bollen et al. further demonstrated the utility of MCTSI in assessing disease severity and the need for intervention [25].

Correlation of MCTSI with clinical and imaging parameters

MCTSI scores showed significant associations with clinical and body-composition variables. Age had no significant impact, whereas male sex and alcohol consumption were associated with higher scores. Patients with local (APFC, ANC) and systemic complications demonstrated significantly elevated MCTSI scores (p < 0.001), indicating strong concordance between imaging severity and clinical outcomes. Higher VAT and VAT/SMT ratios were strongly associated with increased MCTSI scores, highlighting the role of visceral adiposity in disease severity and supporting the value of MCTSI as an effective risk-stratification tool.

Concordance between MCTSI and Revised Atlanta Classification

This study demonstrates strong concordance between MCTSI and the Revised Atlanta Classification, with most mild and all severe cases showing complete agreement (p < 0.001). Minimal discrepancies were observed in the moderate category, supporting the reliability of MCTSI as an imaging-based severity assessment tool. The high concordance underscores the value of integrating imaging and clinical scoring systems for effective prognostication and management. These findings are consistent with studies by Liao et al. and Sahu et al., which reported a significant correlation between MCTSI and APACHE II scores in predicting disease severity and outcomes [23,24].

Limitations

This study has several limitations. Although the sample size was adequate for the primary objectives, it was insufficient to evaluate outcomes such as mortality and ICU admission. VAT is metabolically active, and even small increases may influence disease severity, particularly in lean individuals. The use of WHO Asia-Pacific BMI cut-offs may limit applicability to other populations. Early CT imaging may be constrained by cost and resource availability, potentially affecting timely assessment. Additionally, the single-center South Indian study population may limit the generalisability of the findings.

## Conclusions

This study demonstrates that VAT and the VAT/SMT ratio are significant early predictors of disease severity in AP. CT-based assessment of these parameters enables early identification of high-risk patients and facilitates timely risk stratification when used alongside established scoring systems such as MCTSI and APACHE II. Male patients exhibited higher VAT and a greater likelihood of severe disease, likely influenced by alcohol consumption, while age-related body composition changes did not significantly affect severity scores or pancreatic enzyme levels. MCTSI showed strong concordance with the Revised Atlanta Classification and correlated closely with APACHE II scores, supporting its role as a rapid and reliable imaging-based tool for assessing disease severity. Elevated VAT and VAT/SMT ratios independently identified patients at risk for severe AP, reinforcing their clinical value in early prognostication.
